# Primary mucin-producing urothelial-type adenocarcinoma of the prostatic urethra diagnosed on TURP: a case report and review of literature

**DOI:** 10.1186/1471-2490-14-39

**Published:** 2014-05-22

**Authors:** Elisabeth M Sebesta, Hossein S Mirheydar, J Kellogg Parsons, Jessica Wang-Rodriguez, A Karim Kader

**Affiliations:** 1 Columbia University College of Physicians and Surgeons, 630 W. 168th St, New York, NY 10032, USA; 2 UC San Diego Health System, 200 W. Arbor Drive #8897, San Diego, CA 92103-8897, USA; 3 VA San Diego Healthcare System, 3350 La Jolla Village Dr. (113), San Diego, CA 92161, USA

**Keywords:** Mucin-producing adenocarcinoma, Prostatic urethra, Trans-urethral resection of prostate, Urothelial-type adenocarcinoma, Adenocarcinoma

## Abstract

**Background:**

Mucin-producing urothelial-type adenocarcinoma of the prostatic urethra is extremely rare. These lesions must be differentiated from other mucinous tumors including mucin-producing prostatic adenocarcinoma and metastases from either colonic or bladder primaries.

**Case presentation:**

We report here a case of urothelial-type adenocarcinoma arising from the prostatic urethra. The patient is an 81 year-old man with a history of pT1 urothelial cell carcinoma of the bladder status post trans-urethral resection of bladder tumor (TURBT) who initially presented with irritative lower urinary tract symptoms and mucosuria refractory to Flomax and finasteride. A shared decision was made for the patient to undergo trans-urethral resection of prostate (TURP). At the time of surgery, a papillary tumor emanating from the prostatic urethra was found and no urothelial lesions were noted in the bladder. Pathology of the resected prostatic chips revealed an invasive adenocarcinoma with intestinal-type differentiation that stained positive for CK7, CK20, and villin, but negative for PSA, PSAP, uroplakin, and CDX-2. Colonoscopy was normal and CT scan did not show any evidence of colonic lesions nor visceral or lymph node metastases. Thus, the patient was diagnosed with a primary urothelial-type adenocarcinoma of the prostatic urethra.

**Conclusion:**

Herein we review the literature regarding this unusual entity, and discuss the differential diagnosis, immunohistochemistry, and the importance of correctly identifying this rare tumor.

## Background

The most common mucin-producing adenocarcinoma of the prostate gland is a primary prostatic adenocarcinoma with mucin production [1]. The differential diagnosis includes secondary prostatic lesions of primary colonic or bladder origin and urothelial-type adenocarcinoma arising directly from the prostatic urethra or prostatic ducts. The latter diagnosis is exceedingly rare, with only 18 cases identified in the literature to date [1-4]. Urothelial-type adenocarcinoma of the prostatic urethra is histologically identical to nonurachal adenocarcinoma of the bladder, however it arises from urethritis glandularis or glandular metaplasia of the prostatic urethra [1,5]. We describe a case of primary urothelial-type adenocarcinoma of the prostatic urethra that was initially diagnosed on TURP specimen, with a discussion of its clinical-pathological correlation.

## Case presentation

**Figure 1:**
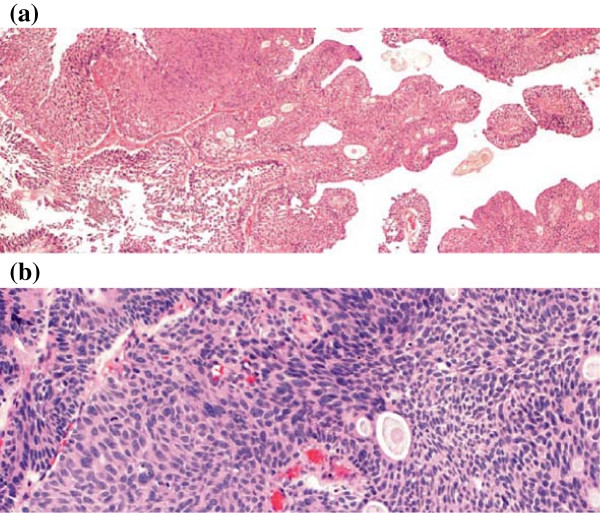
**pT1 urothelial cell carcinoma.** Patient’s original biopsy showing high grade papillary urothelial carcinoma of the bladder. Hematoxylin-eosin stain. **(a)** 40x magnification. **(b)** 100× magnification.

An 81 year-old Caucasian man with past medical history of COPD, on home oxygen, presented with mucosuria, urinary frequency, and nocturia. Digital rectal exam demonstrated a 60 cc benign-feeling gland and the patient’s PSA was 0.38. The patient has a history of a 2.5 cm pT1 urothelial cell carcinoma at the bladder dome status post TURBT 19 months prior (Figure 1a, 1b). The patient had two subsequent urine cytology examinations 5 and 2 months prior, both of which were negative for malignant cells. The patient recently noticed mucosuria. His subsequent surveillance cystoscopy demonstrated a normal-appearing bladder but a fibrinous material emanating from the prostatic urethra and median lobe of the prostate, however no distinct urothelial mass was seen and the bladder urothelium appeared unremarkable. There was evidence of a prostatic adenoma, and the patient was scheduled for TURP via greenlight PVP. During the procedure the prostatic urethra was clearly visualized. The prostatic adenoma seen at the bladder neck was vaporized until the veromontanum was visualized. A white fibrinous material originating from the veromontanum was seen. A prostatic resection was initiated using bipolar TURP. Deep to the fibrinous material, a papillary tumor was visualized. TURP was completed using bipolar without incidence.

### Pathological results

**Figure 2:**
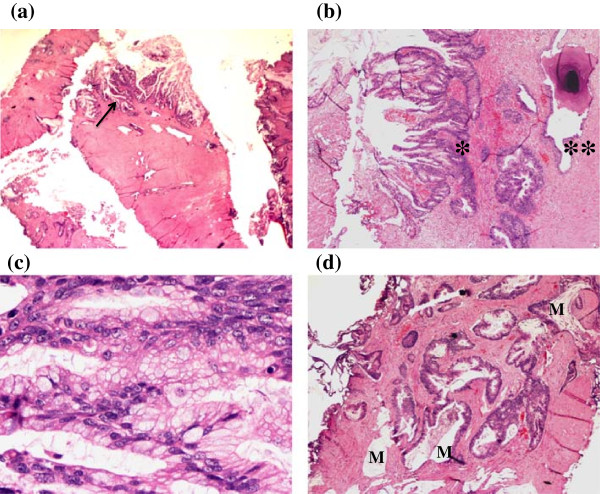
**TURP prostate chip specimen.** Hematoxylin-eosin stain. **(a)** 10× magnification, arrow demonstrates adenocarcinoma of the prostate with complex glandular arrangement. **(b)** 40× magnification, adenocarcinoma (*) at the edge of the TURP fragments which contrasts with nearby benign prostatic glands (**). **(c)** 400× magnification, demonstrating extensive intestinal differentiation. **(d)** 40× magnification, adenocarcinoma with extensive mucin (M) dissecting into the prostatic stroma.

**Figure 3:**
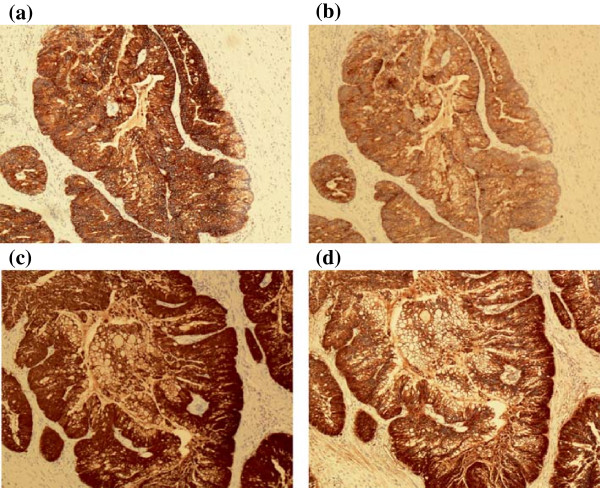
**Surgical specimen and positive pathological staining. (a)** Diffusely positive staining for CK20. **(b)** Diffusely positive staining for CK7. **(c)** Diffusely positive staining for villin. **(d)** Mostly cytoplasmic positive staining for beta-catenin.

**Figure 4:**
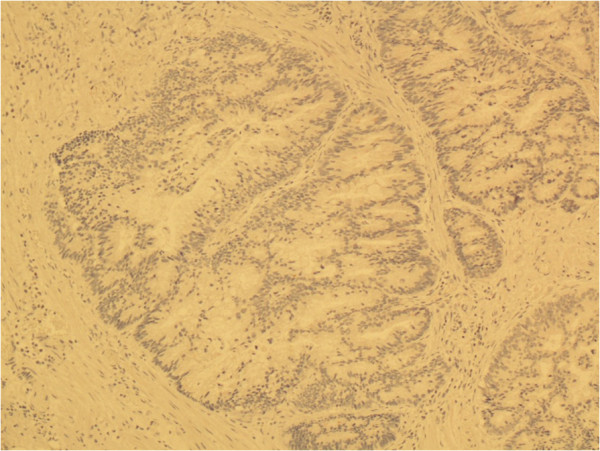
**Surgical specimen and negative pathological staining.** Diffusely negative staining for PSA.

Final pathology revealed intestinal type epithelium in a background of necrosis and inflammatory debris together with invasive adenocarcinoma with extensive intestinal differentiation (Figure 2a-d). The specimens were found to stain strongly positive for CK20, CK7, and villin (Figure 3a-c). Beta-catenin showed strong cytoplasmic and membranous staining (Figure 3d). In addition, the tumors were negative for uroplakin, CDX-2, PSA (Figure 4), and PSAP staining. Final urine cytology contained atypical urothelial cells suspicious for malignancy.

### Follow-up

Due to the histological appearance of the prostatic tumor, the patient underwent a colonoscopy to rule out a primary colonic malignancy. Multiple diverticula were found in the sigmoid colon and six sessile polyps were biopsied from the ascending and descending colon, all of which were found to be benign. In addition the patient underwent a CT urogram, which was without evidence of a filling defect or bladder primary. The patient was thus given the diagnosis of primary adenocarcinoma of the prostatic urethra. He was evaluated for radical cystectomy and was deemed very high risk for operative management due to his severe chronic pulmonary obstructive disease (COPD). He underwent an oncology evaluation including PET CT scan demonstrating no evidence of metastatic disease. Based on this evaluation, the patient declined to undergo exenterative surgery due to concerns for decreased quality of life. A decision was thus made for him to undergo local aggressive trans-urethral resection with adjuvant multimodal therapy including 42 days of Intensity Modulated Radiotherapy (IMRT) and radiosensitizing oral capecitabine chemotherapy (900 mg po qd × 6 weeks). Capeitabine versus a platinum-based chemotherapy was chosen as the patient had several contraindications to platihum-based chemotherapy including hearing loss, CKD, and peripheral neuropathy. His PSA remains low at 0.074.

### Discussion

Mucin-producing urothelial-type adenocarcinoma of the prostate is an exceedingly rare diagnosis, and one that is poorly understood thus far. It is thought to arise from precursor lesions (urethritis glandularis and glandular metaplasia) found in the prostatic urethra or prostatic ducts [1,5]. The published case reports of this disease have helped to determine some of the clinical and pathological features.

In 1996, Tran and Epstein reported the first two cases of mucinous adenocarcinoma arising from the prostatic urethra. In both cases, the patient presented with obstructive urinary symptoms. The gross pathology of the tumors revealed mucin lakes lined by tall columnar epithelium, one specimen of which contained mucin-positive signet ring cells. Both patients had a negative colonoscopy and cystoscopy to rule out bladder and colon primaries, and both had low PSA values. The patients remained without evidence of disease with short-term follow-up following radical retropubic prostatectomy in the first patient and external beam radiotherapy in the second [2].

Curtis, et al. in 2005 described an additional two cases. One man presented with urinary retention, bilateral flank pain, microhematuria, and renal insufficiency and the other with a nodule on digital rectal exam. Again the patients had a low PSA and a negative cystoscopy and colonoscopy work-up. The first patient underwent TURP for bladder outlet obstruction, and shortly after was found have liver metastases and died from disseminated intravascular coagulation. The second patient was treated with radical prostatectomy with bilateral lymph node dissections, and remained with no evidence of disease for 16 months of follow-up [3].

Osunkoya, et al. in 2007 published 15 cases identified between 1990 and 2006, the largest collection to date. All patients presented with an element of urinary obstructive symptoms, however other symptoms included mucosuria (20%), hematuria (13.3%), and urinary tract caliculi (20%). The PSA remained <1.5 in 9/10 patients throughout follow-up. Treatment included TURP in 7/15 and radical pelvic resection in 8/15, including 5 radical prostatectomies (3 of which underwent adjuvant therapy), 2 cystoprostatecomies, and 1 pelvic exenteration. Among these men, 53% died of their disease at an average of 49.2 months from presentation, and all 8 men undergoing radical resection had evidence of extraprostatic extension, 4 of whom developed metastatic disease to the lungs, liver, pelvic side wall, and/or testes. The two men initially described by Tran and Epstein as having no evidence of disease at short-term follow-up both eventually died of their disease at 13 and 63 months [4].

This large collection of cases encompassed only 0.02% of their consult service for prostate cancer. In addition, this description of cases was the first to truly highlight the aggressiveness of the disease with a clinical course that differs drastically from the common adenocarcinoma of the prostate. In these cases, bone metastases were not present at time of progression, and progression of disease to death was more common and rapid [4].

All case reports thus far have helped to identify the histologic features and the immunohistochemical staining that can help differentiate this disease on pathology. Precursor lesions identified in men include glandular metaplasia of the prostatic urethra (53%) and villous features (47%). All specimens contained mucin pools lined by pseudostratified columnar mucinous epithelium with varying degrees of cytologic atypia. Other less commonly demonstrated patterns include necrosis, signet ring cells, and perineural invasion [1-4].

Curtis, et al. identified that in comparison to prostatic adenocarcinoma with mucin production, both urothelial-type adenocarcinoma arising in the prostatic urethra and secondary adenocarcinoma of colonic origin involving the prostate were PSA and PSAP negative and CK20 positive. In addition they proposed that a colonic primary would stain negative for CK7 and 34βE12 (high molecular weight cytokeratin) while a prostatic primary would stain positive [3]. Osunkoya, et al. corroborated these findings, in addition to identifying that 10/15 specimens in their study also stained negative for beta-catenin and CDX-2 [4]. Tamboli, et al. demonstrated that villin staining was useful in differentiating the primary tumor, as it was positive in colonic adenocarcinoma but negative in urothelial carcinoma with glandular differentiation [6]. However villin is also positive in primary enteric-type adenocarcinoma of the urinary tract, and therefore not helpful in distinguishing this diagnosis from colonic metastases.

## Conclusion

Using the work of our predecessors, we were able to diagnose the case reported here with primary mucin-producing urothelial-type adenocarcinoma of the prostatic urethra. We obtained an immunohistochemical profile including PSA, PSAP, CK7, CK20, CDX-2, and villin. This in addition to a negative colonoscopy and CT urogram helped to differentiate between prostatic adenocarcinoma with mucin production from secondary lesions from colon or bladder to suggest a diagnosis of primary urothelial-type adenocarcinoma of the prostatic urethra. As evidenced by previous case reports, making the correct diagnosis in this disease early is crucial. First, it may save the patient multiple invasive procedures and work-up to exclude other primaries. In addition, the distinction from prostatic adenocarcinoma with mucin production is critical, as the disease described here is more aggressive and deadly. It cannot be treated with hormonal therapy and assigning a Gleason score is rendered useless. Continuing to gather data and case reports will be crucial to help determine the clinical behavior and to identify the optimal treatment for this rare and aggressive disease.

### Consent

Written informed consent was obtained from the patient for publication of this case report and any accompanying images. A copy of the written consent is available for review by the Series Editor of this journal.

## Abbreviations

TURP: Trans-urethral resection of prostate; TURBT: Trans-urethral resection of bladder tumor; PSA: Prostate-specific antigen; PSAP: Prostatic-specific acid phosphatase; CK7: Cytokeratin 7; CK20: Cytokeratin 20; CDX-2: Caudal type homeobox 2.

## Competing interests

The authors declare that they have no competing interests.

## Authors’ contributions

EMS wrote the manuscript and made the revisions. JWR performed the pathological evaluation and provided the slides for pathology. HAM, JKP, and AKK treated the patient. HAM obtained written informed consent from the patient for submission of this case report. All authors read and approved the final manuscript.

## Pre-publication history

The pre-publication history for this paper can be accessed here:

http://www.biomedcentral.com/1471-2490/14/39/prepub
